# The insula, a grey matter of tastes: a volumetric MRI study in dementia with Lewy bodies

**DOI:** 10.1186/s13195-020-00645-y

**Published:** 2020-07-06

**Authors:** Nathalie Philippi, Vincent Noblet, Malik Hamdaoui, David Soulier, Anne Botzung, Emmanuelle Ehrhard, Benjamin Cretin, Frédéric Blanc, AlphaLewyMA study group, Catherine Martin-Hunyadi, Catherine Demuynck, Pierre Anthony, Catherine Mutter, Jennifer Kemp, Laetitia Monjoint, Timothée Albasser, Stéphanie Rauch, Clélie Phillipps, Lucie Rauch

**Affiliations:** 1 Neurology Department, Neuropsychology Unit, University Hospital of Strasbourg, Strasbourg, France; 2 CMRR (Memory Resources and Research Centre), University Hospital of Strasbourg, Strasbourg, France; 3 ICube Laboratory (UMR 7357) and FMTS, University of Strasbourg and CNRS, Strasbourg, France; 4 Stroke Unit, University Hospital of Strasbourg, Strasbourg, France; 5 Geriatrics Department, University Hospital of Strasbourg, Strasbourg, France

**Keywords:** Insula, Personal tastes, Personal preferences, Self, Dementia with Lewy bodies

## Abstract

**Background:**

Despite the growing number of discoveries during the past decades about its functions, the insula remains a mysterious ‘island’. In addition to its involvement in basic functions such as gustation and interoception, the insular cortex is now considered a key region for integrated functions such as emotion/motivation processing, decision-making and self-consciousness. We hypothesized that this structure, standing at the crossroads of such functions, could ground personal tastes in general, beyond food preferences and aesthetic judgements. Given that dementia with Lewy bodies is characterized by a focal atrophy within the insular cortex from the early stages, this condition provides an opportunity to test such a hypothesis.

**Methods:**

We developed a questionnaire to assess potential changes in personal tastes, submitted it to a cohort of 23 patients with early-stage dementia with Lewy bodies and compared their questionnaire results to those of 20 age-matched healthy controls. Furthermore, we performed a global and regional neuroimaging study to test for a potential correlation between the patients’ scores for changes in personal tastes and their insular cortex volumes.

**Results:**

Our results indicate that the patients presented significant changes in personal tastes compared to the controls, in both food and non-food domains. Moreover, imaging analyses confirmed the involvement of the insular cortex atrophy in the changes in personal tastes using global analysis, and in both food and non-food domains using regional analysis.

**Conclusions:**

These results bring new insights into the role of the insula as a ‘grey matter of tastes’, this structure supporting personal preferences in general, beyond the food domain. The insular cortex could be involved through its role in motivational processes by the representation of subjective awareness of bodily states during the phenomenological experience of stimulus appraisal. However, we also argue that it could support the abstract representations of personal tastes as self-concepts, acutely exemplifying embodied cognition. Finally, the questionnaire on changes in tastes could constitute an interesting tool to help early diagnosis of dementia with Lewy bodies and to assess insular dysfunction more generally.

## Background

The insular cortex is involved in numerous functions, the complexity of which increases according to the posterior-anterior axis [1] and is sustained by a specific type of large pyramidal neurons uniquely developed in hominoid primates, the Von Economo neurons [2]. Insular functions range from primary functions, such as gustation [3, 4] and interoception [5, 6], to more integrative functions which are based on the formal representations of bodily states, such as self-consciousness [1, 7] and social cognition [8, 9], together pursuing individual homeostasis. By reverberating emotions through viscerosensory responses, the insula plays a central role in the regulation of emotions [10–12] and influences decision-making [13, 14]. Moreover, beyond its role as a primary gustatory cortex, the insula determines food preference, triggering aversion against harmful food and appetite toward beneficial food according to both survival needs and pleasantness [4]. Food appraisal relies on the insula’s connections to the reward/motivation networks (i.e. ventral striatum, orbitofrontal cortex), as is the case for addictions [15] and aesthetic judgements [16], this structure being now integrated in the hedonic system in general [17].

In the present study, we posited that the insular cortex could serve as a support for personal tastes in general, beyond food preferences and aesthetic judgements. For the purpose of this study, we used the word ‘taste’ interchangeably with ‘preference’, to refer to ‘the fact of liking or enjoying something’ (Cambridge dictionary). Across literature [18–22], personal tastes are associated to some consistent characteristics, which could rely on the insular cortex, namely, subjectivity, pleasure/motivation and judgement/decision-making. (i) First, as conveyed by the expression ‘a matter of taste’, personal preferences are characterized by an idiosyncratic subjectivity. From the perspective of cognitive models of the self, if personal preferences belong to the conceptual self [23, 24], they also imply the subjective sense of self inherent in the subject liking or disliking an object at the present moment [18, 19, 21, 22]. (ii) During personal experiences, preferences are positively or negatively reinforced depending on their utility and/or pleasantness through emotion [4, 17, 19, 21]. Such reinforcement learning could derive from the survival needs through evolutionary processes [16], unpleasant experiences being negatively reinforced in the course of one’s personal history, just as the experience of eating food that causes intoxication. Conversely, experiences favouring survival or pleasure are likely to be reinforced. (iii) Finally, motivational processes underlie decision-making, which allows the expression of personal preferences [25]. Standing at the crossroads of self-consciousness, emotional/motivational and decision-making networks [1], the insular cortex therefore appears as a potential key region to underpin personal tastes in general, likely co-opting the ancestral circuitry of food appraisal in the course of human evolutionary development [16]. Other potential anatomical supports of personal tastes are the cortical midline structures, such as the medial prefrontal and medial parietal cortices, which are key regions for self-processing in general and conceptual self in particular [26–29].

The hypothesis that the insular cortex could sustain personal tastes in general is supported by observations of behavioural changes in patients with insular stroke [30]. In that study, three patients had experienced changes in personal tastes after a right insular stroke that were not limited to food preferences, but also involved tastes for dressing and physical appearance. Dementia with Lewy bodies (DLB) provides an opportunity to study progressive insular dysfunction, since it is characterized by the existence of an early atrophy of the insula [31–33]. Furthermore, the presence of Lewy bodies within the anterior insula, including in the Von Economo neurons, has recently been confirmed [34]. To date, changes in personal tastes have never been described in DLB patients, even though they frequently present with anosmia and agueusia [35], which might affect their tastes for food. Beyond the consequences of sensory deprivation, DLB patients could experience changes in food preferences and personal tastes in general, consecutive to insular dysfunction. Modifications such as preference for sweet and binge eating are common features of fronto-temporal lobar degeneration [36] and have already been related to insular atrophy, among other regions involved in reward processes [37–39]. Dysfunction of this region has also been associated with deficits in ‘taste cognition’ in Alzheimer’s disease and vascular dementia [40]. Interestingly, changes in music tastes have additionally been reported in isolated cases of patients with fronto-temporal lobar degeneration [41].

In this context, we designed a questionnaire in order to explore potential changes in personal tastes in a cohort of patients with early stages of DLB and to test a potential relationship with the insular atrophy using volumetric analysis. We included a section for non-food domains, since we were more particularly interested in exploring the potential role of the insula in personal tastes in general, beyond food preferences. From a clinical perspective, the aim was to better understand behavioural changes in DLB patients and to develop a potential clinical tool that could reflect insular damage. More theoretical issues were also raised, and this study also aimed at bringing new insights into the role of the insular cortex in personal tastes in general. We hypothesized that changes in personal tastes would be observed in patients with early DLB both in food and non-food domains, and would be correlated to insular atrophy, among other regions involved in self-concept, such as cortical midline structures.

## Methods

### Population

Twenty-three patients with early-stage DLB and 20 healthy control subjects (CS), who were participating in a larger cohort study, AlphaLewyMA (http://clinicaltrials.gov/ct2/show/NCT01876459), were enrolled in the study. Patients were recruited from the tertiary memory clinic of the University Hospital of Strasbourg, France. Control subjects were recruited from the hospital’s Clinical Investigation Centre. The patient group and the control group were matched for age and educational level (Table 1). Patients had to be in the prodromal or mild stages of the disease (Mini-Mental State Examination [MMSE] score ≥ 20), with probable DLB according to the current and formal criteria [42, 43] and to DSM-5 for the prodromal stage [44]. Thus, in addition to the cognitive impairment, they had to fulfil a minimum of two of the following core criteria for the disease: fluctuations, parkinsonism, and hallucinations. For the purposes of neuropsychological assessment, all participants were required to be fluent in French, have a minimum of 9 years of formal education and have no significant visual or auditory disabilities. Exclusion criteria for all participants included history of alcohol/substance abuse, relevant neurological or psychiatric comorbidities or the presence of other severe or unstable medical illness. Participants with an abnormal neurological examination—except for parkinsonism in the case of patients—depression symptoms (mini-GDS, [45]) or significant cerebral vascular burden (Wahlund scale > 2, [46]) were excluded from the study. Finally, patients with claustrophobia or contra-indication to MRI were excluded. All participants provided written informed consent for the study, in accordance with the Declaration of Helsinki, and the study was approved by the local Ethics committee of East France (IV).

**Table 1:** Demographic data of the DLB and control groups

Demography/cognitive measures	DLB group (***n*** = 23)	Control group (***n*** = 20)	Student’s ***t*** test or chi-squared test
**Age**	74.0 (9.6)	72.0 (6.1)	*t* = 0.78, *P* = 0.44
**F/M**	16/7	13/7	*χ*^2^ = 0.10, *P* = 0.75
**Education level (years)**	12.3 (3.9)	11.5 (3.2)	*t* = 0.69, *P* = 0.50
**MMSE score**	25.9 (2.6)	28.5 (1.0)	*t* = − 4.25, *P* < 0.001

*DLB* dementia with Lewy bodies, *F/M* female/male ratio, *MMSE* Mini-Mental State Examination

### Behavioural study

#### Cognitive assessment

Participants underwent a full cognitive evaluation, that was described in a previous study [47]. Memory was assessed using the French version of the Free and Cued Selective Reminding Test [48] and the Delayed Matching to Sample-48 items [49]. Executive functions we evaluated using the Frontal Assessment Battery [50], letter fluency [51] and Trail Making Test B [52]. Processing speed was assessed using the Trail Making Test A [52] and the digit symbol substitution test [53]. Among tests evaluating instrumental functions, Rey-Osterrieth Complex Figure test [54] was used to assess visuoconstructive abilities and lexical fluency to assess semantic evocation [51]. Patients were impaired in every domain compared to controls (Table 2).

**Table 2:** Cognitive measures of the DLB and control groups

Cognitive measures	DLB group (***n*** = 23)	Control group (***n*** = 20)	Student’s ***t*** test
**MMSE score**	25.9 (2.6)	28.5 (1.0)	*t* = − 4.25, *P* < 0.001
**FCSRT (score/48)**	39.5 (6.8)	45.9 (3.04)	*t* = − 3.74, *P* < 0.001
**DMS-48 (score/48)**	41.9 (7.5)	47.1 (1.1)	*t* = − 3.00, *P* = 0.002
**FAB (score/18)**	14.9 (2.6)	16.9 (1.1)	*t* = − 2.93, *P* = 0.003
**Letter fluency**	16.5 (6.7)	23.3 (6.2)	*t* = − 3.33, *P* < 0.001
**TMT B (s)**	172.0 (80.1)	92.4 (26.6)	*t* = 4.10, *P* < 0.001
**TMT A (s)**	98.2 (105.6)	43.7 (11.6)	*t* = 2.23, *P* = 0.02
**Digit symbol (score/19)**	7.8 (3.7)	11.4 (2.8)	*t* = − 3.42, *P* < 0.001
**Lexical fluency**	22.8 (7.7)	36.4 (6.5)	*t* = − 5.95, *P* < 0.001
**ROCF (score/36)**	28.5 (9.4)	33.9 (2.0)	*t* = − 2.39, *P* = 0.01

*DLB* dementia with Lewy bodies, *MMSE* Mini-Mental State Examination, *FCSRT* Free and Cued Selective Reminding Test (sum of total recall TR1, TR2, TR3), *DMS-48* Delayed Matching to Sample-48 items (Set2), *FAB* Frontal Assessment Battery, *TMT* Trail Making Test, *ROCF* Rey-Osterrieth Complex Figure test

#### Changes in personal tastes

The questionnaire on changes in personal tastes comprised 10 questions designed to assess different domains: gustatory alterations, changes in food preferences, tobacco/alcohol use, clothes, music/art, decoration, leisure activities (i.e. questions 1 and 2 ‘Do you feel that food is less tasty/tastes differently than before?’; questions 3 and 4 ‘Have you, without any particular reason, started/stopped eating something that you didn’t use to like/used to like before?’; question 5 ‘Have you, without any particular reason and without any feeling of lack, stopped smoking or drinking alcohol,?’; question 6 ‘Have you, without any particular reason, feel like wearing clothes that you wouldn’t have worn before?’; question 7 ‘Have you, without any particular reason changed tastes for music or art?’; question 8 ‘Have you, without any particular reason, feel like to change the decoration of you house (furniture, painting)?’; question 9 and 10 ‘Have you, without any particular reason, started/stopped any leisure activities that you didn’t use to like/used to like before?’). The expression ‘without any particular reason’ was included in each question in order to avoid bias related to potential modifications due to causes other than changes of tastes, such as disabilities. Patients were instructed to consider changes in personal tastes occurring over recent years. Each question was read aloud by the examiner and participants were invited to evaluate the extent to which they agreed with the statement according to a 5-point scale that was presented visually on a double-headed arrow. The more the response reflected changes in tastes, the higher the score (4 = ‘fully agree’; 3 = ‘rather agree’, 2 = ‘neither agree nor disagree’, 1 = ‘rather disagree’, 0 = ‘fully disagree’), with a maximum total score of 40. Moreover, for each item, patients were given the opportunity to exemplify their changes in tastes, so that the examiner could prompt them not to take into account modifications for reasons other than changes of tastes (e.g. ‘I stopped cycling because my balance was not so good’). Since we wanted to investigate personal tastes in general, beyond food preferences, we also considered a non-food score after excluding the four questions that were related to gustation and food preferences, with a maximum score of 24.

#### Anosmia/agueusia

Patients were also asked whether they had anosmia or agueusia, which could influence changes in tastes toward food. The patients’ scores were therefore compared between those who reported anosmia or agueusia, or both, and those who reported neither.

### Statistical analysis for the behavioural study

Student’s *t* test was used to compare intergroup differences between DLB patients and control subjects for both demographic and behavioural quantitative data. For the behavioural data, a one-tailed *t* test was used, since we hypothesized that the DLB patients would have higher scores than the controls due to more pronounced changes of tastes. Given the small sample size of the DLB patient’s group with anosmia or agueusia, a Mann-Whitney *U* test was used to compare the scores of changes in tastes between the DLB patient’s groups with or without anosmia or agueusia. A chi-squared test was used to compare the sex ratio between groups. A threshold of *P* < 0.05 was used to determine statistical significance. Pearson’s test was used for correlation analyses to verify if behavioural data such as changes in tastes would be independent from demographic/cognitive measures.

### Imaging study

We used both voxel-based morphometry (VBM) and region of interest (ROI) analyses to investigate the neuroanatomical correlates of potential changes in personal tastes in the DLB patients. Each participant underwent a high-resolution MRI scan, within 6 months of neuropsychological testing. High-resolution anatomical images were obtained with a 3T MAGNETOM Verio (Siemens Healthcare, Erlangen, Germany) equipped with a 32-channel Siemens head coil. The acquisition included a 3DT1-weighted MPRAGE sequence (TR = 1900 ms, TI = 900 ms, TE = 2.53 ms, FA = 9°, GRAPPA = 2, voxel size = 1 mm^3^).

#### VBM analysis

VBM analyses included image pre-processing and statistical analyses. These steps were carried out using the SPM12 software package (Wellcome Department of Imaging Neuroscience, London; http://www.fil.ion.ucl.ac.uk/) running on Matlab R2017 (MathWorks, Natick, MA, USA). Anatomical MRI images were spatially preprocessed using standard procedures [55]. All T1-weighted structural images were first segmented, bias-corrected and spatially normalized to the Montreal Neurological Institute (MNI) space using an extension of the unified segmentation procedure [56] that includes six classes of tissue. The DARTEL registration toolbox was then used to build a study-specific template and to bring into alignment all of the segmentation images. The VBM analysis was done on modulated grey matter (GM) images; that is, the GM value in each voxel was multiplied by the Jacobian determinant derived from the spatial normalization. This procedure preserves the total amount of GM from the original images. These modulated GM images were smoothed with a Gaussian kernel (full width at half maximum [FWHM]: 8 mm). To map the regions of atrophy related to the potential changes in personal tastes, we tested correlation between the GM volume at a voxel level and both the total and non-food scores of the questionnaire on changes in tastes using the general linear model. Correlation analysis between behavioural data and GM volume was performed using a threshold of *P* = 0.001 uncorrected, including age and total GM (TGM) as nuisance covariates. Experiments were also conducted while considering the MMSE as an additional covariate to investigate the potential impact of disease severity. Only negative correlations were considered, since we hypothesized that significant alterations of taste (i.e., higher scores on the questionnaire) would be related to lower insular volume. A cluster spatial extent of 50 voxels was used to avoid irrelevant and isolated detections. The software Xjview (http://www.alivelearn.net/xjview8/) allowed us to characterize each cluster.

#### ROI analysis

**Fig. 1:**
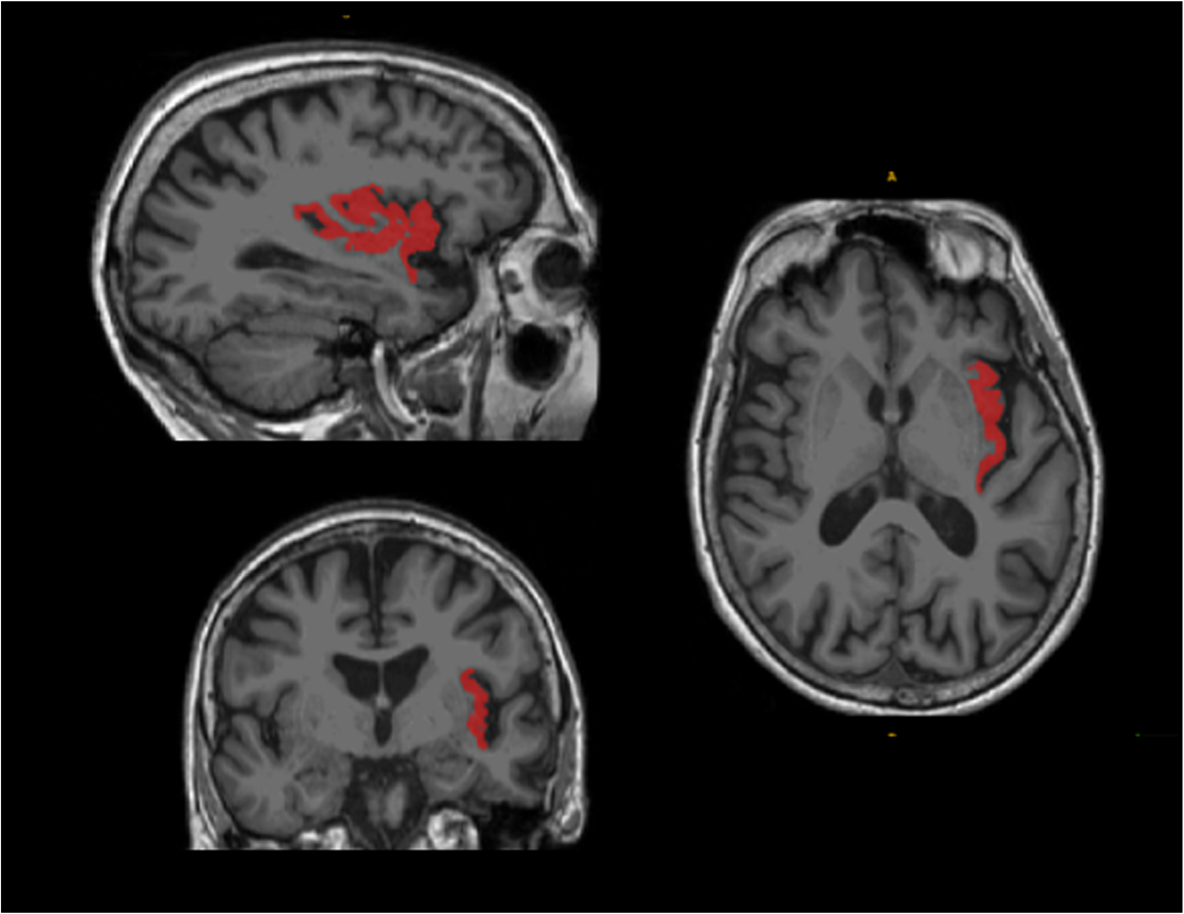
Triplanar view of the right insular cortex using the ROI analyses with Freesurfer

Insular cortex volumes (Fig. 1) were extracted in the left and the right hemisphere using Freesurfer (http://surfer.nmr.mgh.harvard.edu/). Correlation analyses between insular volumes and behavioural data were carried out using a general linear model, with age and TGM as nuisance covariates, with the StatsModels python module (http://statsmodels.sourceforge.net/), using a statistical threshold of *P* = 0.05. As in the VBM study, only negative correlations were considered, on the assumption that higher scores would be associated with more pronounced atrophy within the insular cortex. A more complex regression model involving additional explanatory variables, namely the MMSE score, the educational level and the volumes of the regions found outside the insular cortex with the VBM analyses (i.e. superior frontal gyrus and rostral anterior cingulate, as labelled in Freesurfer), was also tested in order to investigate their potential contributions.

## Results

### Behavioural study

#### Changes in tastes

The behavioural results indicated that the DLB group disclosed significant changes in personal tastes compared to the CS group, both for the total scores (mean 8.5, SD 5.6 vs. 4.3, SD 4.5, *t* = 2.6, *P* = 0.006), for the food scores (mean 4.04, SD 3.81 vs. 1.65 SD 2.21; *t* = 2.5; *P* = 0.009) and for the non-food scores (mean 4.4., SD 3.1 vs. 2.7, SD 2.9, *t* = 1.9, *P* = 0.03, see Table 3). On qualitative analysis, when considering answers 3 and 4, changes were distributed as follows: 27% of gustatory alterations, 20% of changes in food preferences, 13% of changes in tobacco/alcohol use, 13% of changes in tastes for clothes, 2% of changes in music tastes, 7% of changes in tastes for decoration and 18% of changes in tastes for leisure activities.

**Table 3:** Scores on the questionnaire on changes in tastes for the DLB and control groups

Changes in tastes	DLB group (***n*** = 23)	Control group (***n*** = 20)	Student’s ***t*** test
**Total (/40)**	8.5 (5.6)	4.3 (4.5)	*t* = 2.6, *P* = 0.006
**Food (/16)**	4.04 (3.81)	1.65 (2.21)	*t* = 2.5, *P* = 0.009
**Non-food (/24)**	4.4 (3.1)	2.7 (2.9)	*t* = 1.9, *P* = 0.03

*DLB* dementia with Lewy bodies

Note that we found no consistent correlations between the scores of changes in tastes and demographic/cognitive measures. Only a correlation was found between the total scores of changes in personal tastes and letter fluencies (*r* = 0.43; *P* = 0.05), and between the non-food scores and the digit symbol scores (*r* = 0.56; *P* = 0.01). These results would not remain significant after a correction for multiple analyses given the number of correlations tested (*n* = 24).

#### Anosmia/agueusia

Six out of the 23 patients disclosed anosmia or agueusia or both. Their scores for changes in tastes were not significantly different from those of the patients who had no such symptoms (mean 9.3, SD 6.7 vs. 8.2, SD 5.4; *U* = 43, *P* = 0.60).

### Imaging study

#### VBM analyses

##### Total score

**Fig. 2:**
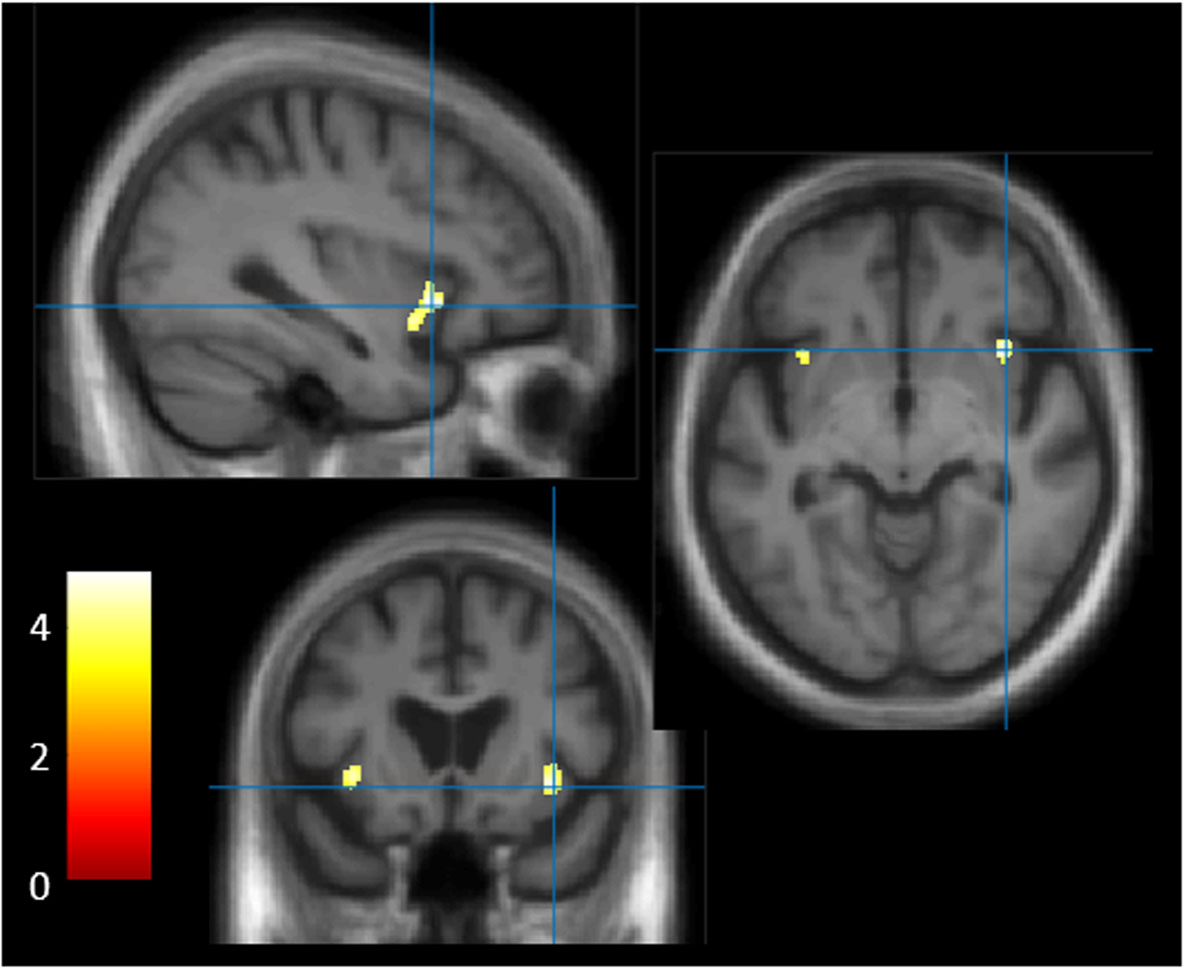
VBM analyses for changes in tastes (total score) in the DLB group. GM volumes within bilateral insular cortices negatively correlated with the total score on the questionnaire on changes in tastes, using a threshold of *P* = 0.001 uncorrected, including age and TGM as nuisance covariates, *k* = 50

VBM analyses for changes in tastes, using the total score in the DLB group, revealed negative correlations to GM regions within the bilateral insular cortex using a threshold of 0.001 uncorrected, including age, and TGM as nuisance covariates, with a minimum cluster size of *k* = 50 (see Fig. 2 and Table 4). Note that including MMSE as an additional nuisance covariate did not significantly change the results (results not shown). Moreover, VBM analyses for the food score brought similar results, though with a prominent involvement of the right insular cortex (Supplemental figure).

**Table 4:** VBM results for the total score on the questionnaire on changes in tastes in the DLB group

VBM	BA	***k***	***x***	***y***	***z***	***T***
**L insula**	13/47	128/133	−34.5	16.5	− 4.5	4.36
**R insula**	13/47	119/155	36	16.5	− 7.5	4.81

*L* left, *R* right, *BA* Brodmann area, *k* cluster size in voxels (specific region’s volume/cluster’s global volume), *x*, *y*, *z* Talairach coordinates, *T* T-value

##### Non-food score

**Fig. 3:**
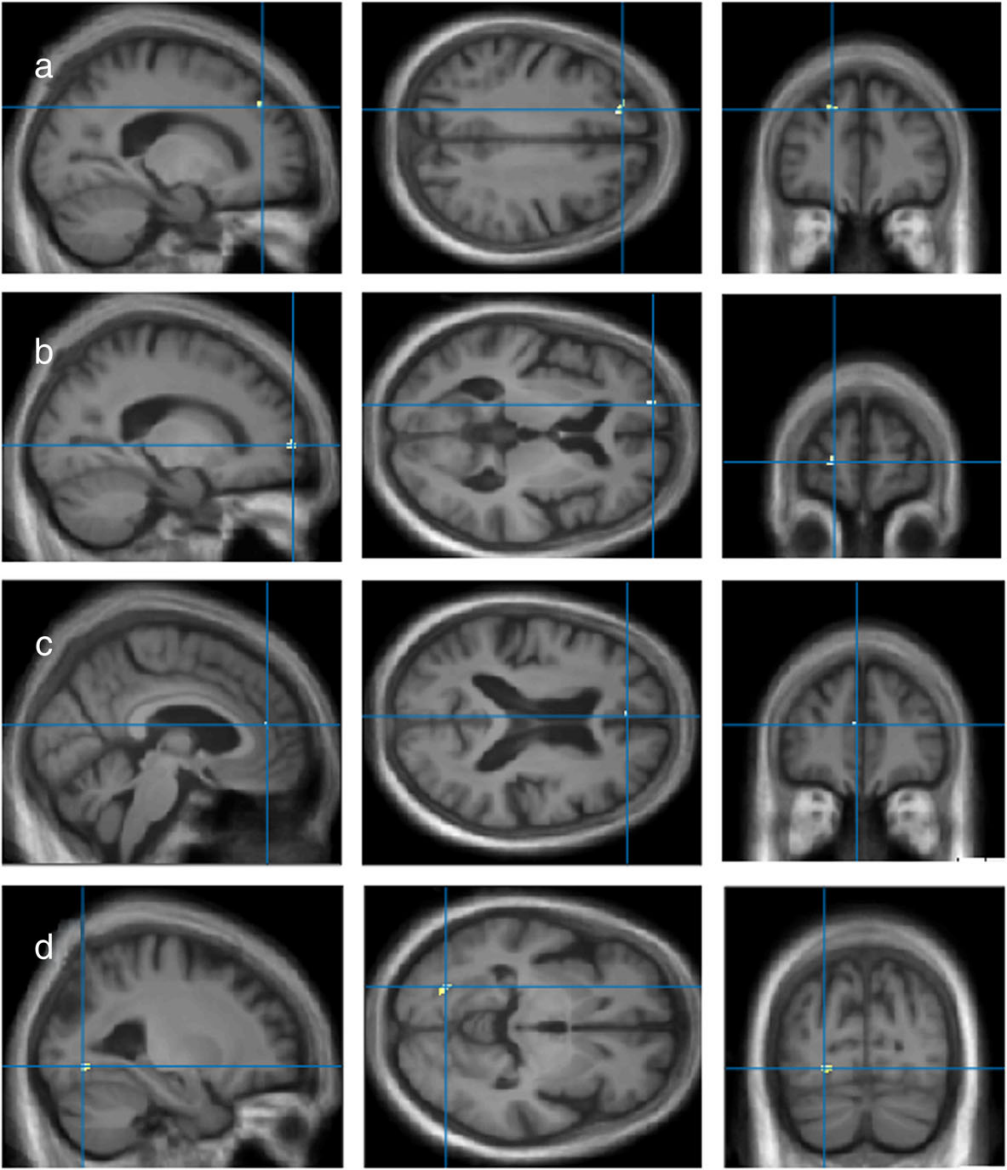
VBM analyses for changes in tastes (non-food score) in the DLB group. GM volumes within the left superior frontal gyrus (**a**, **b**), anterior cingulate (**c**) and occipito-temporal region (**d**) negatively correlated with the non-food score on the questionnaire on changes in tastes, using a threshold of *P* = 0.001 uncorrected, including age and TGM as nuisance covariates

VBM analyses for changes in tastes using the non-food score in the DLB group did not reveal any correlation using a threshold of *P* = 0.001 uncorrected, including age, and TGM as nuisance covariates, with a minimum cluster size of *k* = 50. Note that when considering smaller cluster sizes, no correlation with the insular cortex was found either. However, other GM regions were negatively correlated with the non-food score on the questionnaire on changes in tastes, including the superior frontal gyrus and the anterior cingulate (BA9 and BA10). Another cluster was found within the occipito-temporal region and was also negatively correlated with the non-food scores, though it mostly included white matter (see Fig. 3 and Table 5). Note that when including the MMSE as an additional nuisance covariate, only the correlation to the left superior frontal gyrus remained significant (results not shown).

**Table 5:** VBM results for the non-food score on the questionnaire on changes in tastes in the DLB group

VBM	BA	***k***	***x***	***y***	***z***	***T***
**L sup. frontal g.**	10	25/25	− 19.5	60	3	3.94
**L sup. frontal g.**	9	30/30	− 19.5	40.5	37.5	3.87
**L ant. cingulate**	10	7/7	− 4.5	43.5	16.5	3.65
**L lingual/fusiform g.**	–	30/38	− 27	− 75	− 6	4.33

*L* left, *R* right, *sup.* superior, *ant*. anterior, *g.* gyrus, *BA* Brodmann area, *k* cluster size in voxels (specific region’s volume/cluster’s global volume), *x*, *y*, *z* Talairach coordinates, *T* T-value

#### ROI analyses with the insular cortex volumes

**Fig. 4:**
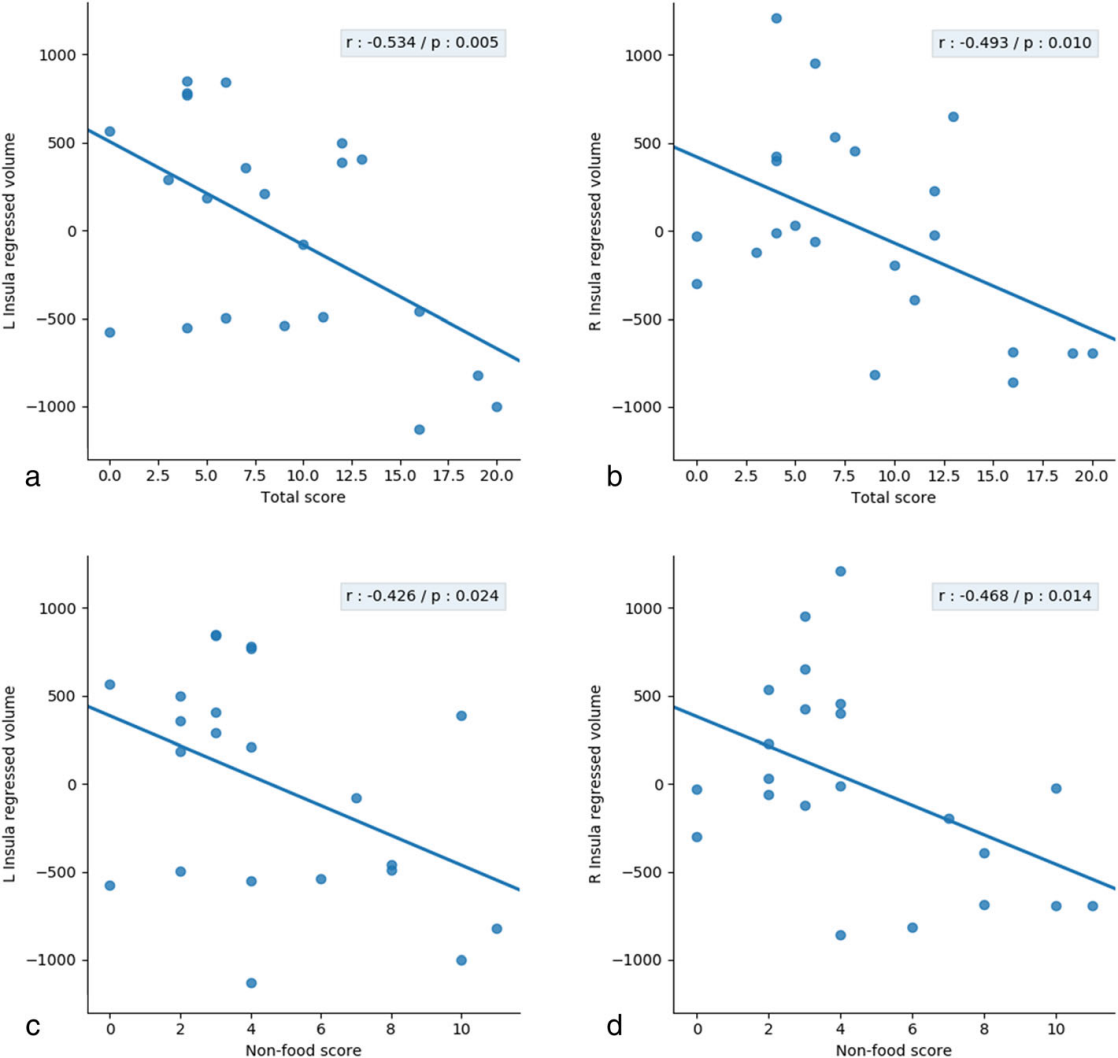
ROI analyses for changes in tastes in the DLB group. Scatterplots showing negative correlations between scores on the questionnaire on changes in tastes and bilateral insular volumes in the DLB group with ROI analyses, using a threshold of *P* = 0.05, including age and TGM as nuisance covariates (panels **a** and **b** show results for the total score, panels **c** and **d** for the non-food score; panels **a** and **c** for the left insular cortex volume, panels **b** and **d** for the right insular cortex volume)

Correlations between the scores on the questionnaire on changes in tastes and bilateral insular cortex volumes were tested in the 23 patients, including age and TGM volume as nuisance covariates. Table 6 shows the results of the correlation analyses and scatterplots are shown in Fig. 4. Negative correlations with the insular cortex volumes were found bilaterally for both the total score (*P* = 0.005 for the left and *P* = 0.01 for the right insular cortex) and the non-food score (*P* = 0.024 on the left and *P* = 0.014 on the right side) on the questionnaire on changes in tastes. Note that when adding the MMSE score and the educational level within the regression analyses, only the MMSE score appeared to have a significant effect on the non-food score (*P* = 0,013 for the left insular cortex; *P* = 0.005 for the right insular cortex). Using this model, the volumes of the insular cortex remained significantly correlated to both the total score (*P* = 0.013 on the left side; *P* = 0.018 on the right side) and non-food score (*P* = 0.019 on the left side; *P* = 0.004 on the right side). Then, the volumes of the anterior cingulate (region labelled ‘rostral anterior cingulate’ in Freesurfer) and of the superior frontal gyrus were considered in the regression model, in addition to age and TGM, in order to test their respective contribution to the non-food score, since these regions were found in the VBM analyses. Only the volume of the superior frontal gyrus appeared to have a significant effect on the non-food score (*P* = 0.04) when testing the model for the right insular cortex. Using this model, correlation to the volumes of the insular cortex remained significant on the right side (*P* = 0.03), but not on the left side (*P* = 0.10).

**Table 6:** Correlations between the changes in tastes and the volume of the insular cortices in DLB group

ROI analyses	Left insula volume	Right insula volume
**Total score**	*r* = − 0.53; *P* = 0.005	*r* = 0.49; *P* = 0.01
**Non-food score**	*r* = − 0.43; *P* = 0.024	*r* = 0.47; *P* = 0.014

## Discussion

In summary, our results indicate that patients with early-stage DLB presented significant changes in tastes compared to age-matched healthy controls, in both the food and non-food domains, independently of the existence of anosmia or agueusia. These results are original regarding DLB and dementias more generally. Concordant with our hypothesis, imaging analyses confirmed the involvement of anterior insular cortex atrophy in the changes in personal tastes. Despite the lack of significant results for the non-food score using VBM analyses, which might be due to a relatively small sample size, ROI analyses confirmed the involvement of the insular cortex for both the food and non-food domains. These results bring new insight into the role of the insular cortex in personal tastes in general, beyond food preferences.

Regarding DLB patients, our study is the first to highlight the existence of changes in personal tastes associated with this disease. Moreover, we confirmed that these modifications were linked to the insular atrophy that occurs early in the course of the disease. Changes in tastes with regard to food are characteristic of fronto-temporal lobar degeneration [36] and have already been linked to the atrophy in this structure, among other regions associated with reward processes [37, 38]. To ensure that the changes in tastes in the DLB patients were not triggered solely in the food domain, we also explored the non-food scores separately and observed that the changes in tastes remained significant compared to the healthy controls when excluding the food domain. Moreover, we verified that the patients’ scores were not influenced by the presence or absence of anosmia or agueusia [35]. Modifications of personal preferences beyond food have seldom been explored in dementias. Only isolated cases of changes in tastes for music in patients with fronto-temporal lobar degeneration have been reported so far [41]. Our results, as well as the observations of changes in personal tastes secondary to insular stroke [30], suggest that such a questionnaire could be an interesting tool to help early diagnosis of DLB and to assess insular dysfunction more generally.

Our results bring new insights regarding the role of insular cortex in personal tastes in general, beyond food preference and aesthetic judgement, since we found insular cortex atrophy to be associated with changes in personal tastes, for food and non-food domains. In addition to its role of gustatory cortex [3], the insular cortex is also regarded as a homeostatic system to guide feeding behaviour, involving not only the perception of taste quality and intensity of food, but also the affective value and pleasantness associated with food [4]. This function is based on reinforcement learning and recruits the same reward circuits as in addictions [15], allowing the experience of a given food to be positively or negatively reinforced, depending on the outcome for the individual. The activity of the insula is also triggered by food pictures [57], which places this structure as an integrated system for food preference independent of the gustatory experience. The insula has also been associated with aesthetic appraisal. Indeed, the right anterior insula was the region most consistently involved in artwork and other stimuli appraisal in a meta-analysis performed across four sensory modalities [16], in addition to the left insula and regions of the reward circuits (ventral striatum, orbito-frontal cortex). The insula therefore appears as a key region to determine personal preferences for food, objects and other stimuli through multimodal phenomenological experience. Lateralization could be influenced by the modality [16] or by the valence associated to the stimuli [58], both perspectives being in agreement with the bilateral involvement in our study, since our questionnaire examines changes in personal tastes independently of the valence and the modality. The fact of liking or disliking an object would emerge from subjective awareness of changes in internal bodily states in the presence of the given object [16], according to Craig’s model [5]. In the same vein, the DLB patients’ changes in tastes might be interpreted as the consequence of modifications in their phenomenological experience toward a stimulus due to insular atrophy. Overall, our results suggest that personal tastes in general, including leisure activities, are supported by the insula, concordant with the view that the basic circuitry used for homeostatic needs has evolved into a pleasure system [17].

Aside from the role of the insula in phenomenological experiencing that is generally emphasized across studies to explain personal preferences [4, 16], we posit that this structure could nonetheless support abstract representation of personal tastes. As a matter of fact, the activation of the insula appears across all modalities [16], and is additionally elicited by supramodal representations such as food names [59] and autobiographical memories [60, 61]. Similarly, correlations to anterior insular volume in our study were obtained with a questionnaire interrogating the *concepts* of the subjects’ tastes. This is coherent with the existence of an increasing level of integration of insular functions across its antero-posterior axis, the posterior and mid insula being involved in basic functions such as interoception and gustation, while the anterior insula would support meta-representation of subjective awareness [1]. Such meta-representations would consist of abstract representations of personal preferences in the present study, the colocalization of which, within the same structure that triggers contextual appraisal through the representation of bodily states, constitutes a relevant illustration of embodied cognition [62]. More generally within the domain of self-concepts, the insula appears to be involved in both self-agency [63] and self-recognition triggered by perception, such as images [64, 65], as well as in evaluation of supra-modal self-descriptive cues such as adjectives or sentences [65–67]. Overall, we suggest that this structure might support self-concepts in general, as is the case for cortical midline structures [26–29]. Contrary to what we expected, no strong correlations were found to the medial prefrontal cortex in association with the changes in tastes using VBM, but only small-sized clusters within the anterior cingulate and superior frontal gyrus. This might be due to the fact that DLB patients have prominent insular atrophy during such early stages.

Our study entails some limitations such as a relatively small sample size and the use of a homemade questionnaire. These preliminary results should therefore be confirmed with additional studies, involving a larger cohort of DLB patients and other clinical populations (e.g. fronto-temporal lobar degeneration, insular stroke), as well as multimodal imaging studies, in order to highlight the networks associated to insular involvement.

## Conclusions

Our study has both clinical and theoretical interests. From a clinical point of view, this study is the first to demonstrate the existence of changes in tastes in DLB patients and in dementias more generally, in both the food and non-food domains. Concordant with our hypothesis, these changes in personal preferences are related to insular atrophy, which develops early in the course of the disease. This finding brings new insights regarding the general role of the insula as a ‘grey matter of tastes’, beyond food and aesthetic appraisal. Moreover, our results suggest that the insular cortex could support personal tastes as abstract representations and not only in terms of phenomenological experience, acutely exemplifying embodied cognition by the representation of self-concepts and bodily self within the same structure.

## Supplementary information

**Additional file 1 : Supplemental figure.** VBM analyses for changes in tastes (food scores) in the DLB group. GM volumes within bilateral insular cortices negatively correlated with the total score on the questionnaire on changes in tastes, using a threshold of *P* = 0.001 uncorrected, including age and TGM as nuisance covariates, *k* = 50.

**Additional file 2.**

**Additional file 3.**

## Data Availability

The datasets analysed during the current study are available from the corresponding author on reasonable request.

## Abbreviations

DLB: Dementia with Lewy bodies
VBM: Voxel-based morphometry
ROI: Region of interest
