# Supplementary figures and images for: The insula, a grey matter of tastes: a volumetric MRI study in dementia with Lewy bodies
Source: Alzheimers Res Ther. 2020 Jul 6;12:79. doi: 10.1186/s13195-020-00645-y (PMC7336457; doi:10.1186/s13195-020-00645-y)

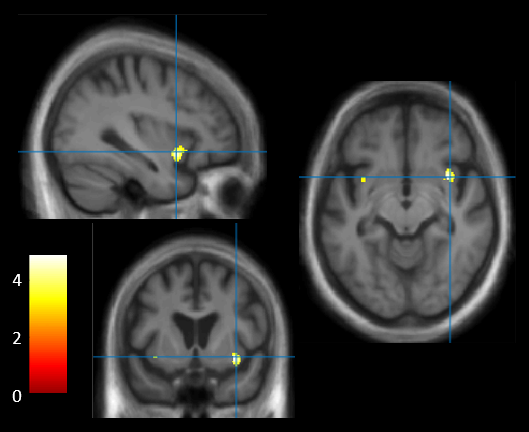

Supplement: Supplementary file 1 — Additional file 1 : Supplemental figure. VBM analyses for changes in tastes (food scores) in the DLB group. GM volumes within bilateral insular cortices negatively correlated with the total score on the questionnaire on changes in tastes, using a threshold of P = 0.001 uncorrected, including age and TGM as nuisance covariates, k = 50. [file 13195_2020_645_MOESM1_ESM.tif]

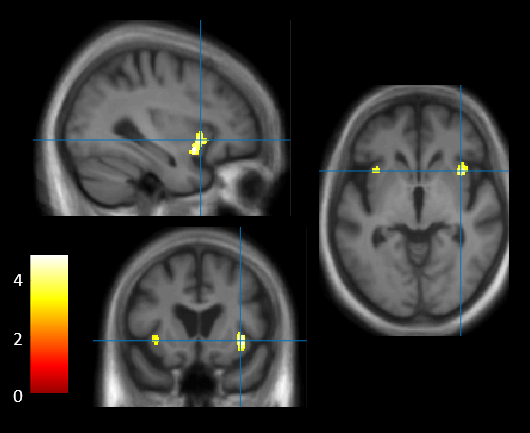

Supplement: Supplementary file 2 — Additional file 2. [file 13195_2020_645_MOESM2_ESM.tif]

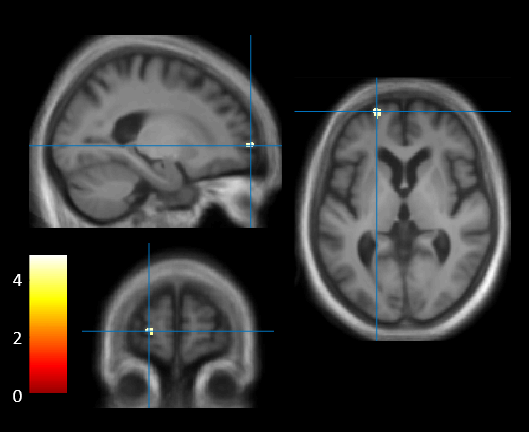

Supplement: Supplementary file 3 — Additional file 3. [file 13195_2020_645_MOESM3_ESM.tif]
